# Spectrum of Opportunistic Infections and Risk Factors for In-Hospital Mortality of Admitted AIDS Patients in Shanghai

**DOI:** 10.1097/MD.0000000000003802

**Published:** 2016-05-27

**Authors:** Bin Luo, Jianjun Sun, Rentian Cai, Yinzhong Shen, Li Liu, Jiangrong Wang, Renfang Zhang, Jiayin Shen, Hongzhou Lu

**Affiliations:** From the Wenzhou Medical University (BL, J-YS, H-ZL), Wenzhou, Zhejiang; Department of Infectious Disease (BL, J-JS, R-TC, Y-ZS, LL, J-RW, R-FZ, J-YS, H-ZL), Shanghai Public Health Clinical Center, Fudan University; and Department of Infectious Disease (H-ZL), Huashan Hospital Affiliated to Fudan University, Shanghai, China.

## Abstract

To investigate the frequency and the spectrum of major opportunistic infections (OIs), evaluate the major clinical factors associated with each specific OI, and identify the risk factors for in-hospital death among HIV patients in East China.

A retrospective cohort study was made including all the HIV-infected patients who were admitted for the first time to the Shanghai Public Health Clinical Center during June 1, 2013 to June 1, 2015. The demographic and clinical data were collected. Comparison of continuous variables was analyzed by one-way ANOVA and rank sum test. Person *χ*^2^ test and Fisher exact test were applied to analyze the categorical variables. A Cox proportional hazards regression model was used to determine the risk for the occurrence of in-hospital death.

In total, 920 patients were enrolled with age of 41.59 ± 13.36 years and 91% male. Median CD4 was 34 (IQR, 13–94) cells/μL. Among these patients, 94.7% acquired OIs while the rest developed malignancies. Pneumocystis pneumonia and bacterial coinfection (42.1%) was found to be the most common OIs, followed by tuberculosis (31.4%), CMV (20.9%), Cryptococcosis (9.0%), and MAC infection (5.2%). Of the above 5 major OIs, CMV-infected patients had the lowest median CD4 cell count 22.50 (IQR, 7.50–82.00) while the patients with tuberculosis infection had the highest count 61.00 (IQR, 27.00–176.00). In-hospital death rate was 4.2 per 100 person-years among these patients. Of note, admitted patients with 2 types of OIs (2.20, 95% CI 1.39–3.48) and those patients who were 40-year old or older (1.75, 95% CI 1.10–2.78) had a higher risk of such death.

Pneumocystis pneumonia and tuberculosis were still the leading causes for the admission of HIV-infected patients in East China, and these patients tended to have very low CD4 cell counts. It is believed that expanding the HIV screening test and pushing the infected ones get ART earlier is required for generating a more successful HIV management strategy.

## Abstract 2

Supplemental Digital Content is available in the text

## INTRODUCTION

According to the China Global AIDS Response Progress Report, 501,000 cases of people were reported to be living with HIV/AIDS by the end of 2014, of whom 205,000 were reported to be AIDS patients in China. Meanwhile, 159,000 deaths had been reported across the country.^1^ HIV infection destroys mainly the adaptive immune system, which makes the affected people highly susceptible to opportunistic infections (OIs) as well as AIDS-related malignancies. Global evidence has shown that OIs grossly affect the health and quality of life of HIV-infected people by causing higher morbidity and mortality among those individuals. Since combined antiretroviral therapy (cART) gradually restores the immune system, its widely use has been associated with a dramatic decreasing of the incidence of OIs in HIV-infected patients.^2–4^

However, AIDS-defining diseases, including OIs and malignancies, still pose a challenge to the healthcare system in China even if the Chinese government initiated the National Free Antiretroviral Treatment Program (NFATP) in 2003.^5^ On the one hand, considering it has been over 2 decades since the first case of HIV infection was found in 1985, an increase in the number of HIV-infected people with an advanced stage of disease is not unexpected. On the other hand, in a relative resource-limited country like China, there is no doubt that not every HIV-infected person is covered by the NFATP to receive ART before they are presenting to hospital with certain HIV-related clinical symptoms. What is more, some of the HIV-infected people always hesitate to receive the antiretroviral therapy after they were diagnosed. Consequently, most of them get admitted because of severe opportunistic infections.^6^

Several studies have described the spectrum of OIs in hospitalized HIV-infected patients in many countries, such as Gabon,^7^ South Africa,^8^ India,^9^ Thailand,^10^ Bangladesh,^11^ South Korea,^12^ and Japan.^13^ Unfortunately, data on the prevalence of various OIs and malignancies in admitted HIV-infected individuals in China is still lacking.^14^

Therefore, the purpose of this study is: to estimate the proportion of admissions attributable to specific OIs among people living with HIV (PLWH) and identify the most frequent ones, to characterize the major clinical factors associated with each specific OI, to determine the cumulative proportion of deaths that occur in hospital of admitted patients and identify risk factors for such mortality.

## METHODS

### Ethics Statement

This research protocol has been approved by the Shanghai Public Health Clinical Center Ethics Committee. Individual written informed consent was waived as the study was retrospective, anonymous and only used the currently existing data.

### Study Design

**FIGURE 1:**
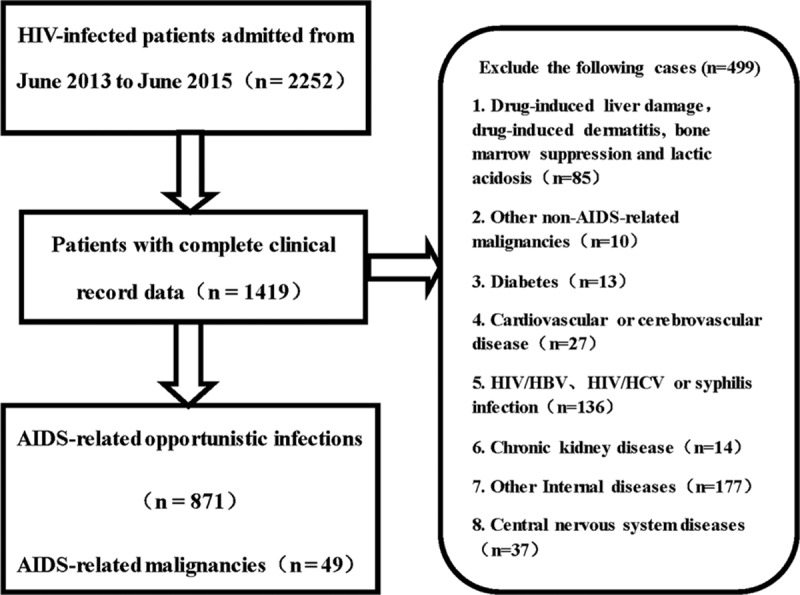
Flow diagram of the study.

This retrospective cohort study was conducted at the Shanghai Public Health Clinical Center, the largest referral center for patients with HIV infection or AIDS in East China. Eligible patients who were for their first time admitted to our center from June 1, 2013 to June 1, 2015 were selected. The inclusive criteria: with HIV antibody test positive (by western blot); with age more than 16-year old. The diagnosis of OIs and malignancies was taken in accordance with the guideline recommended by the United States Center for Disease Control and Prevention (CDC).^15^ CMV viremia was detected by PCR. Patients with other non-AIDS-related opportunistic infections and malignancies or with other internal diseases were excluded: drug-induced liver damage, drug-induced dermatitis, bone marrow suppression, and lactic acidosis (n = 85); other non-AIDS-related malignancies (n = 10) ; diabetes (n = 13); cardiovascular or cerebrovascular disease (n = 27); HIV/HBV,HIV/HCV or syphilis infection (n = 136); chronic kidney disease (n = 14); other Internal diseases (n = 177); central nervous system diseases (n = 37) (Figure 1). The demographic and clinical information including age, sex, duration of ART before admission, length of hospital stay, treatment outcomes, geographic distribution, and occupations and medical insurance were collected. CD4 levels and HIV viral load were measured within 1 week after admission.

### Statistical Analysis

Entry and analysis of all data were conducted using IBM SPSS version 19.0 (IBM SPSS Inc, Armonk, NY). Mean (± standard deviation, SD) was used to describe continuous variables that meet the normal distribution, while percentage (%) was used for categorical variables. When statistical distribution was skewed, data were expressed as median (interquartile range (IQR)). Comparison of continuous variables was analyzed by 1-way ANOVA or Kruskal–Wallis test or Mann–Whitney test. Person *χ*^2^ test and Fisher exact tests were applied to analyze the categorical variables. A Cox proportional hazards regression analysis was made to determine the risk for the occurrence of in-hospital death. Factors significantly associated (*P <*0.10) in univariate analysis were included in multivariate analysis. Statistical significance was determined using a conventional *P* level of 0.05 (2-tailed). All of the clinical data underlying these findings are listed in the Supplementary Material File.

## RESULTS

### Characteristics of the Study Population

In total, 1419 patients were admitted to the Department of Infectious Diseases of Shanghai Public Health Clinical Center from June 1, 2013 to June 1, 2015. A total of 920 patients, of whom 871 had OIs and 49 had HIV-related malignancies, were selected. The rest 499 patients with other clinical symptoms were excluded from this study (Figure 1).

Of the 920 patients whose medical records were analyzed, their clinical characteristics were summarized as shown in Table 1. These patients came from different provinces in China, with 800 (87.0%) being from East China, followed by 44 (4.8%), 37 (4.0%), 17 (1.8%), 16 (1.7%), and 6 (0.7%) of them from Southwest, Central, Northeast, Northwest, and North of China.

**TABLE 1:**
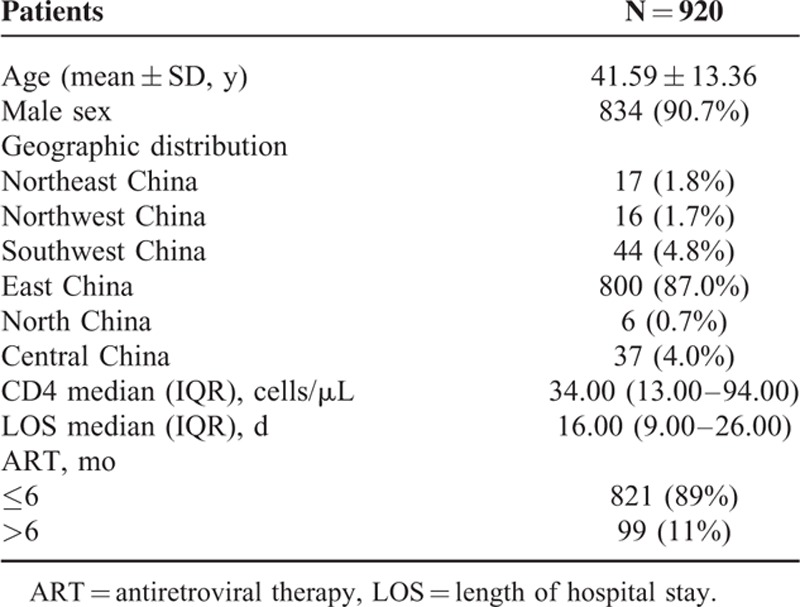
Clinical Characteristics of Selected Patients

Eight hundred thirty-four (91%) were male, and the mean (± SD) age was 41.59 (± 13.36) years. The median (IQR) CD4 cell count, which was detected within 1 week after admission, was 34 (13–94) cells/μL, with 560 (60.9%) patients having a CD4 count less than 50 cells/μL, followed by 142 (15.4%), 114 (12.4%), 70 (7.6%), and 34 (3.7%) of the patients having a CD4 count of 51 to 100, 101 to 200, 201 to 350, and more than 350 cells/μL, respectively. Six hundred sixty-nine (72.7%) patients paid to take a HIV viral load test on admission. The HIV-RNA median log_10_ copies/mL of these patients was 5.35 (IQR, 4.97–5.70). Of note, 821 (89%) patients had received ART for no more than 6 months before presenting to our hospital, including 666 patients who were treatment naive. Among the 254 patients (27.6%) who had received ART before admission, 222 (24.1%) were on a regimen with 2 types of nucleoside reverse transcriptase inhibitors (NRTIs) and 1 type of non-nucleoside reverse transcriptase inhibitors (NNRTI), 30 (3.3%) were with 2 types of NRTIs and 1 type of protease inhibitors (PIs), 1 (0.1%) was with 2 types of NRTIs and 1 type of integrase inhibitors (INIs), and another was with 1 NRTI + 1 NNRTI + 1 PI. The median (IQR) length of stay in the hospital was 16.00 (9.00 – 26.00) days.

### Prevalence of Major OIs and Its Related Factors

Of the total 920 clinical records analyzed, 871 (94.7%) had opportunistic infections (OIs), and only 49 (5.3%) had AIDS-related malignancies (Table 2). Among the patients who acquired OIs, 845 patients received anti-infection treatment before ART initiation, while AIDS-related OIs newly occurred in 75 patients who were on ART. The most common OIs were Pneumocystis pneumonia (PCP) and bacterial coinfection, and the prevalence was 42.1%. This was followed by tuberculosis (31.4%). CMV was the third most frequent OIs and developed in 20.9% of patients: retinitis, 42 patients; viremia, 148 patients; pneumonia, 1 patient; and colitis, 1 patient. The frequencies of other OIs among 920 patients are: Cryptococcosis (83 patients, 9.0%), Mycobacterium avium complex (MAC) infection (48 patients, 5.2%), Penicillium Marneffei infection (43 patients, 4.7%), Herpes zoster (33 patients, 3.6%), Cerebral Toxoplasmosis (17 patients, 1.8%), progressive multifocal leukoencephalopathy (PML) (4 patients, 0.4%), and other fungal infections (46 patients, 5.0%). Kaposi sarcoma (16 patients, 1.7%) and lymphoma (34 patients, 3.7%) were emerged as common AIDS-related malignancies. Diffuse large B-cell lymphoma (12 patients, 1.3%) was the most common malignancies seen among patients with lymphoma, which was followed by unclassified lymphoma (10 patients, 1.1%), Bunkitt lymphoma (10 patients, 1.1%), and plasmablastic lymphoma (2 patients, 0.2%).

**TABLE 2:**
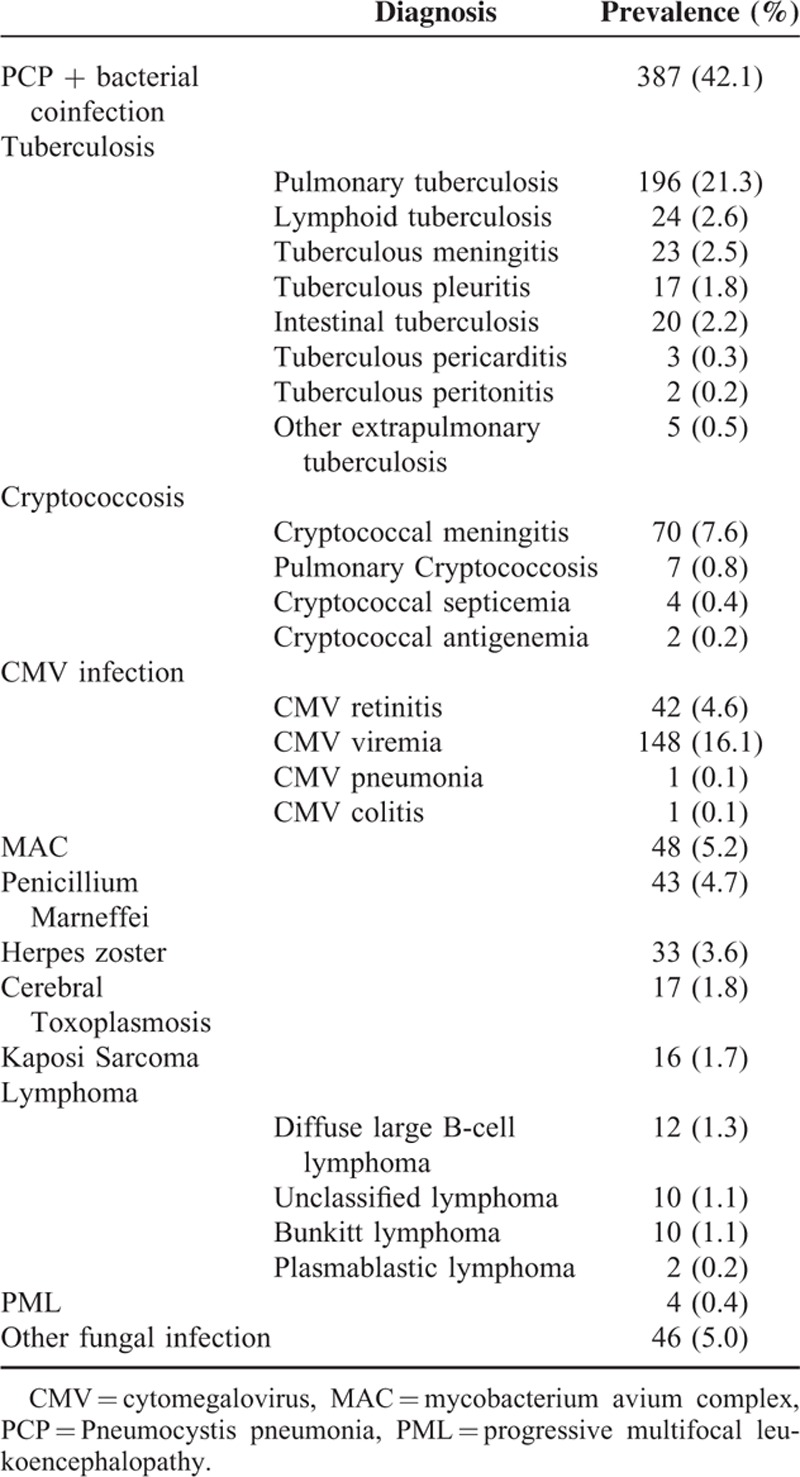
Prevalence of Opportunistic Infections and AIDS-Related Malignancies in 920 Patients

In addition, among the major 6 groups with different OIs, the clinical factors including CD4 cell count, length of hospital stay, and the duration of ART before admission were statistically different (*P* <0.05) (Table 3). However, the other demographic factors such as age, sex, geographic origin, or the outcome (death or discharge) after treatment were not significantly different among the groups with different OIs. Of the 6 etiologic groups with different infections, CMV-infected patients had the lowest median CD4 cell count 22.50 (IQR, 7.50–82.00) while the patients with tuberculosis (TB) infection had the highest CD4 cell count 61.00 (IQR, 27.00–176.00). Notably, the group of people with PCP plus bacterial infections had the highest in-hospital mortality rate (7.2%), while the 32 patients infected by MAC were all survived after treatment.

**TABLE 3:**
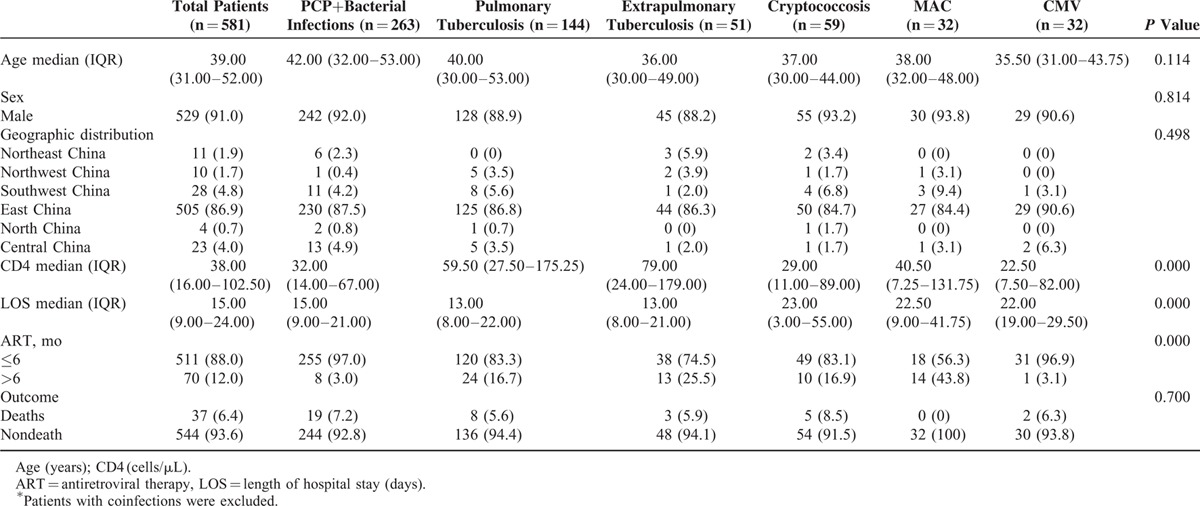
Comparisons Among the Hospitalized HIV Patients With Different Opportunistic Infections^∗^

### Risk Factors for Hospital Death in HIV-Infected Patients

**FIGURE 2:**
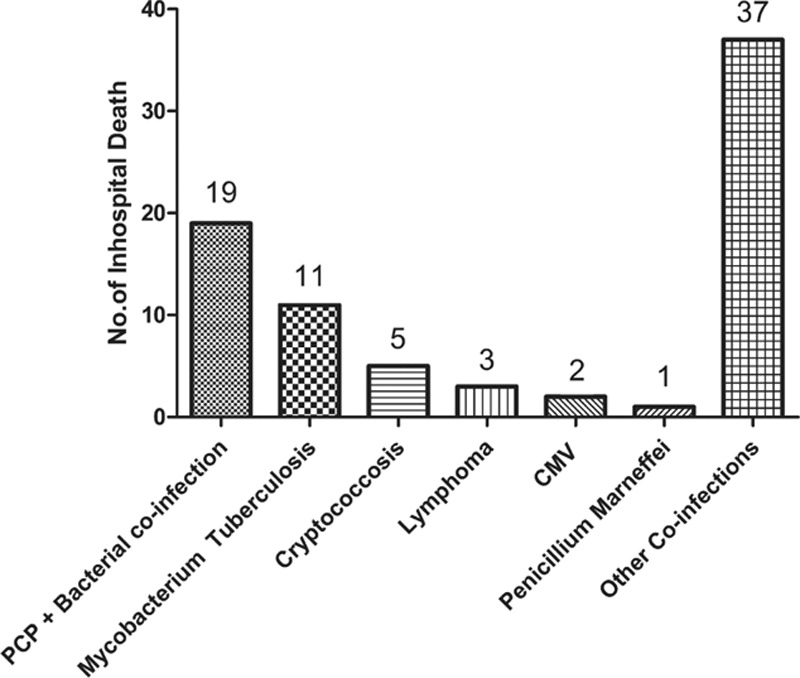
Incidence of in-hospital death caused by specific type of opportunistic infections.

Among 920 patients who admitted due to OIs and AIDS-defining malignancies, 78 patients (8.5%) died while being treated in our hospital. The underlying causes of death of patients before being discharged are depicted in Figure 2. Of note, nearly half of the patients died in hospital due to other coinfections (47.4%). The second most prevalent in-hospital mortality cause was PCP and bacterial coinfections, followed by Mycobacterium Tuberculosis, Cryptococcosis, Lymphoma, CMV infection, and Penicillium Marneffei infection.

In the univariate analysis, the mean age (±SD) is significantly higher in the group of patients who died in hospital (47.38 ± 13.66 vs. 41.01 ± 13.30, *P* <0.001). The median CD4 cell count was significantly lower in the group of patients who died on admission (19.50 vs. 36.00, *P* <0.01). There was no significant difference between the duration of ART before hospitalization of the 2 groups (*P* = 0.20) (Table 4). In addition, the distribution of various types of AIDS-related diseases (OIs and cancers) was also not significantly different between the patients who died or survived in our hospital (*P* = 0.95). However, according to the results from multivariate Cox regression analysis, neither the CD4 cell count (*P* <0.16) nor the ART duration before admission (*P = *0.633) was the risk factor for in-hospital death. Interestingly, patients who were 40-year old or older had almost a 2-fold (AHR = 1.746; 95% CI, 1.097–2.777) higher risk of mortality while still being hospitalized compared with those less than 40-year old. Likewise, admitted patients with 2 types of OIs were about 2 times (AHR = 2.199; 95% CI, 1.389–3.480). (Table 5).

**TABLE 4:**
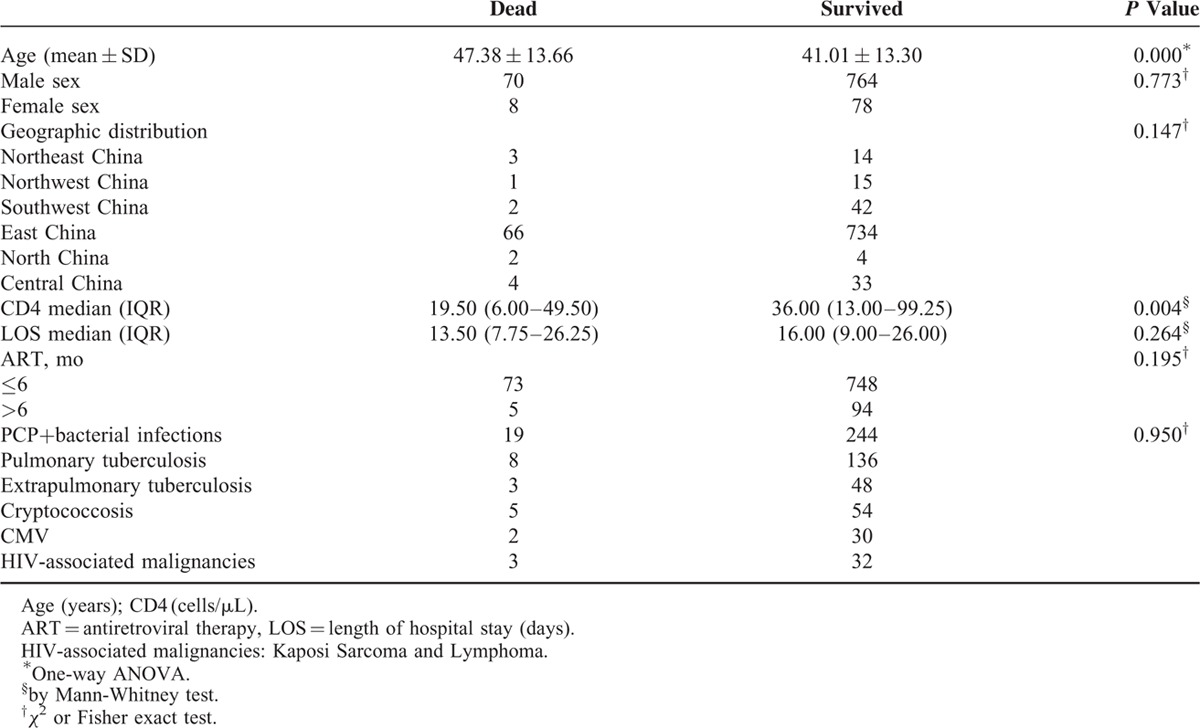
Univariate Predictors of In-Hospital Death in HIV-Infected Patients

**TABLE 5:**
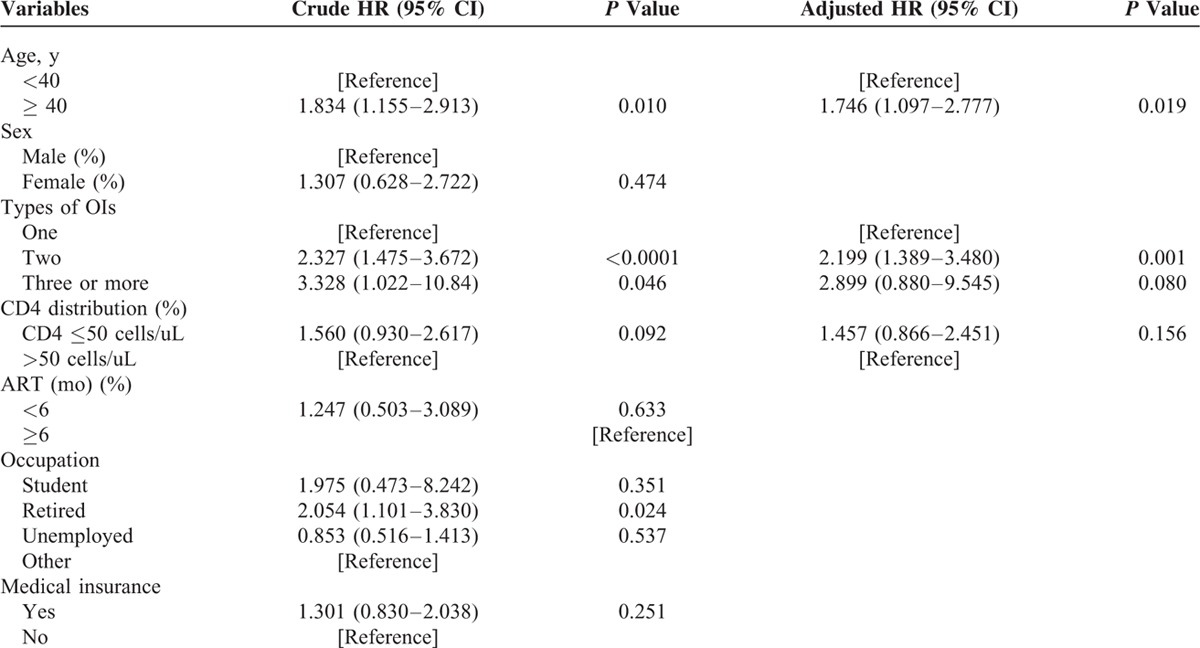
Risk Factors for In-Hospital Death Among HIV Patients at Shanghai Public Health Clinical Center, Shanghai, China, 2013–2015

## DISCUSSION

Despite the implementation of NFATP for more than 1 decade,^5,14,16^ the number of hospitalized HIV-infected patients due to OIs is still high. In China, OIs as well as HIV-related malignancies were treated in referral HIV/AIDS centers of tertiary care hospitals mainly located in big cities of each province. Therefore, a systemic evaluation of the nationwide prevalence and spectrum of OIs and HIV-related cancers in Chinese HIV-positive individuals is still lacking due to limited access to diagnostic and surveillance data. A recent retrospective observational study was conducted in Ditan hospital,^14^ but the spectrum of OIs and malignancies reported mainly applies to the North China.

In this study, the frequency and types of major OIs and malignancies in HIV-infected patients hospitalized at Shanghai Public Health Clinical Center were assessed. This hospital is the major center for HIV/AIDS diagnosis and treatment for people living in the East coastal area of China. Consistently, 800 of the 920 enrolled patients (87%) came from the East China. Hence, the results generated from this study could neither represent the whole patients nor be applied to other regions of China.

As a result, 94.7% of the patients acquired OIs, and only 5.3% of them developed AIDS-related malignancies. Notably, 91% of the affected patients were male, which is consistent with the findings in Ditan hospital of China (82.8%), India (91.2%), and Iran.^14,17,18^ In addition, the (mean ± SD) age of the affected patients was (41.59 ± 13.36) years, which indicates HIV-related diseases occurred more in the economically productive age group. This will probably increase the economic burden of their family and also our country.

Bacterial pneumonia and PCP coinfection was the most prevalent OIs in HIV patients in our study, as shown in other HIV-transmission categories,^19^ and followed by tuberculosis. Considering many studies in resource-limiting areas all observed that tuberculosis was the commonest OIs, our finding was unanticipated. One reason for this incompatible observation we made is because there actually is no consensus on the diagnostic criteria for PCP.^20^ Although an elevated serum level of lactate dehydrogenase (LDH) or S-adenosylmethionine (SAM) has been suggested as surrogate markers for PCP, these clinical findings are not specific enough to distinguish PCP from other types of pneumonia.^21,22^ Another reason is that the patients with PCP had a very low CD4 level when admitted, so it was very likely that these patients had an additional bacterial infection.

Tuberculosis was the second most prevalent OIs in HIV-infected patients in this study, and the prevalence was 31.4%. This observation matches with many other findings.^9,12,14,18,23–25^ However, the frequencies of other major OIs were somewhat different. Previous studies that were conducted in Ditan hospital of China and in developed countries have shown that MAC infection was the common OI.^14^ In contrast, a relative low prevalence of MAC infection was observed in our study.

In this study, we did not list the diagnosis of Candidiasis because this kind of fungi infection is prevalent among HIV-infected patients in advanced stage, especially when their CD4 cell count is less than 50 cells/μL.^26,27^ However, most of our patients with this kind of infection receive treatment in the clinic but only a few patients get admitted.

The overall in-hospital mortality rate was 4.2 per 100 person-years, and the patients presented with other coinfections had the highest mortality rate (47.4%). Notably, none of the 32 hospitalized patients with MAC infection died before being discharged from our hospital. More importantly, we found that patients with age ≥40-year old, or with 2 types of OIs were at higher risk for death in hospital. Unexpectedly, CD4 cell counts <50 cells/μL (AHR of 1.457, 95% CI, 0.866–2.451, *P* = 0.16) was not a risk factor for predicting in-hospital death. It is consistent with another study in Botswana which also showed that there was no correlationship between lower CD4+ T-cell count and early mortality after ART.^28^ This phenomenon may also be explained by the endowed limitations in the present study. First, this is a retrospective study that is simply based on the data deposited in a tertiary referral HIV/AIDS care center, so the observations made in this study are undoubtedly somewhat biased. Second, almost all of the patients had a very low CD4 cell count on admission, so it is not impossible that certain number of patients have died before arriving at the hospital. Last but not least, the endpoint of study is restricted to the in-hospital period, which means the full access to patients’ follow-up clinical data after being discharged would not always be available.

In conclusion, PCP and TB were still the leading causes for the admission of HIV-infected patients in East China, and these patients tended to have very low CD4 cell counts. Being diagnosed and initiating ART timely should shrink the number of HIV patients with severe OIs, therefore, expanding the HIV screening test and pushing the infected ones get ART earlier is the keystone for generating a more successful HIV management strategy.

## Supplementary Material

Supplemental Digital Content
